# Incorporating Within-Host Diversity in Phylogenetic Analyses for Detecting Clusters of New HIV Diagnoses

**DOI:** 10.3389/fmicb.2021.803190

**Published:** 2022-02-17

**Authors:** August Guang, Mark Howison, Lauren Ledingham, Matthew D’Antuono, Philip A. Chan, Charles Lawrence, Casey W. Dunn, Rami Kantor

**Affiliations:** ^1^Center for Computational Biology of Human Disease, Brown University, Providence, RI, United States; ^2^Center for Computation and Visualization, Brown University, Providence, RI, United States; ^3^Research Improving People’s Lives, Providence, RI, United States; ^4^Division of Infectious Diseases, The Alpert Medical School, Brown University, Providence, RI, United States; ^5^Division of Applied Mathematics, Brown University, Providence, RI, United States; ^6^Department of Ecology and Evolutionary Biology, Yale University, New Haven, CT, United States

**Keywords:** HIV, cluster inference, profile sampling, phylogenetics, next generation sequencing (NGS), near-whole-genome, consensus sequence, transmission disruption

## Abstract

**Background:**

Phylogenetic analyses of HIV sequences are used to detect clusters and inform public health interventions. Conventional approaches summarize within-host HIV diversity with a single consensus sequence per host of the *pol* gene, obtained from Sanger or next-generation sequencing (NGS). There is growing recognition that this approach discards potentially important information about within-host sequence variation, which can impact phylogenetic inference. However, whether alternative summary methods that incorporate intra-host variation impact phylogenetic inference of transmission network features is unknown.

**Methods:**

We introduce *profile sampling*, a method to incorporate within-host NGS sequence diversity into phylogenetic HIV cluster inference. We compare this approach to Sanger- and NGS-derived *pol* and near-whole-genome consensus sequences and evaluate its potential benefits in identifying molecular clusters among all newly-HIV-diagnosed individuals over six months at the largest HIV center in Rhode Island.

**Results:**

*Profile sampling* cluster inference demonstrated that within-host viral diversity impacts phylogenetic inference across individuals, and that consensus sequence approaches can obscure both magnitude and effect of these impacts. Clustering differed between Sanger- and NGS-derived consensus and *profile sampling* sequences, and across gene regions.

**Discussion:**

*Profile sampling* can incorporate within-host HIV diversity captured by NGS into phylogenetic analyses. This additional information can improve robustness of cluster detection.

## Introduction

HIV continues to be a significant cause of morbidity and mortality in the United States (US) (Fauci and Lane, 2020). Public health officials and providers are interested in inferring transmission links between individuals with HIV to inform and improve treatment and prevention approaches (Hogben et al., 2016). In the absence of reliable patient contact histories, phylogenetic analysis of HIV sequence data can and has been used to infer transmission clusters (Leitner et al., 1996), under the assumption that two individuals sharing a most recent common ancestor in a phylogeny are more likely to share or lead to an epidemiological link in the real, unobservable transmission network. The application of molecular epidemiology and cluster inference techniques in public health interventions to disrupt transmission was delineated as one of the four key pillars for ending the HIV epidemic in the US (Fauci et al., 2019).

While historically phylogenetic informativeness of the HIV *pol* genomic region was suggested and contested (Hué et al., 2004; Stürmer et al., 2004), its use is now widespread in cluster inference, often due to the availability of sequences from guideline-recommended routine drug resistance testing, typically performed by commercial Sanger sequencing (Panel on Antiretroviral Guidelines for Adults and Adolescents, 2020). In a recent review of HIV cluster inference, 98 out of 105 (93%) analyzed the *pol* region (Hassan et al., 2017).

The increasing availability of NGS technology has led to longer (across more genes) and deeper (multiple reads that correspond to multiple within-host genomes) sequencing of HIV, and data sets more routinely cover nearly the whole genome at great depth (Voelkerding et al., 2009). Recent evidence suggests improvements in both phylogenetic analysis and cluster inference from longer near-whole-genome HIV sequences obtained with NGS. For example, Yebra et al. (2016) found that the accuracy of phylogenetic reconstruction and cluster inference on simulated sequences improved with longer genomic regions (with the best accuracy from a *gag*-*pol*-*env* concatenation). Novitsky et al. (2015) similarly studied effects on cluster inference of using longer genomic regions from near-whole-genome publicly available Sanger sequences and found that the proportion of sequences in clusters increased with longer sequences. Even before the availability of NGS, using longer regions of the HIV genome was shown to improve phylogenetic reconstruction. In one of the earliest studies of HIV sequence data with a known HIV transmission network, Leitner et al. (1996) found that combining data from the *gag* and *env* regions improved the accuracy of phylogenetic reconstruction.

While potential advantages of longer NGS sequences in inferring clusters have been examined (Novitsky et al., 2015; Yebra et al., 2016), advantages of deeper sequencing are less investigated, and whether it can improve HIV molecular clustering inference is unknown. This is due to limitations in established practices of inferring HIV phylogenies across hosts. Researchers often rely on a single consensus sequence for each host that discard all but the majority variant at each site, since most molecular epidemiology approaches require a single fully-resolved sequence per individual in the phylogeny. Accordingly, researchers studying HIV transmission networks discard available information on within-host variation, known to impact phylogenetic inference (Leitner et al., 1996; Leitner, 2019).

The consensus approach, which to date has been employed with Sanger sequencing data in multiple studies of HIV molecular epidemiology (Hassan et al., 2017), carries an underlying statistical assumption of *low relative entropy* (Guang et al., 2016). For HIV, this is equivalent to the strong assumption that a consensus sequence adequately captures all relevant information about HIV diversity within an individual and that variation within hosts has no information about relationships across hosts. While many researchers understand that this assumption is likely wrong and intra-host variation is relevant for phylogenetic analysis of HIV [for a recent review, see Leitner (2019)], in practice researchers have faced limitations in data collection that prevent measuring intra-host variation or in available analysis methods that preserve intra-host variation during alignment and phylogeny. With the advent of long read sequencing technologies for full HIV genomes, obtaining fully resolved sequences that represent the within-host viral population will be possible, but methods to incorporate intra-host variation for transmission cluster analysis will still need to be developed.

Two previous studies have accounted for within-host variation in deeply-sequenced NGS data with coalescent evolutionary models (Romero-Severson et al., 2014; Giardina et al., 2017), but such models still assume a consensus sequence as the observed data. Two other studies introduced methods to use deeply-sequenced HIV data without assuming a consensus, for a different but related epidemiological goal of estimating transmission directionality and identifying multiple infections (Skums et al., 2018; Wymant et al., 2018). Methods also exist that combine haplotype estimation from deeply-sequenced NGS data and phylogenetics (Bendall et al., 2021) as a way to incorporate within-host diversity, but available haplotyping methods have a high computational cost and results are often not sufficiently accurate for cluster analysis (Wymant et al., 2018). Additionally, all aforementioned methods that do not rely on a consensus incorporate within-host diversity by including multiple sequences or tips per sample, which presents difficulties with summarizing or collapsing the resulting phylogenetic tree in order to identify transmission clusters and measure cluster certainty.

In this study, we develop a new method we call *profile sampling* that incorporates within-host HIV genome variation into phylogenetic analyses used to identify transmission clusters. We examine if, and to what extent, incorporation of within-host variation available from deeply-sequenced Illumina-based NGS data provides improved phylogenetic inference and clustering relative to traditional consensus-sequence-based approaches. We focus our analyses on all newly HIV-diagnosed individuals during six months from the largest HIV center in Rhode Island, US.

## Materials and Methods

### Data Collection and Sequencing

HIV-1 *pol* Sanger sequences (HXB2 positions 2253-3554), available through clinical care, were collected from the 37 adults (18 years) newly-diagnosed with HIV-1 during the first six months of 2013 and treated at The Miriam Hospital Immunology Center in Providence, Rhode Island, US. Patients at this Center represent ∼80% of the state’s HIV epidemic.

In addition, blood was obtained from consenting participants and processed to isolate RNA from plasma (*n* = 27), and proviral DNA from whole blood (*n* = 4) or peripheral blood mononuclear cells (PBMC; *n* = 6). Using Sanger sequencing and Illumina-based NGS, near-whole-genome viral sequences were obtained from one compartment; plasma for participants with detectable viral load and proviral DNA for participants with undetectable viral load or unsuccessful plasma genotyping. The study was approved by the Institutional Review Board at Lifespan, which is the parent health network of The Miriam Hospital.

Total nucleic acids were extracted and an in-house genotyping assay was used to generate the near-whole genome sequence (wgs), based on previously published methods (Nadai et al., 2008; Di Giallonardo et al., 2014). For each sample, two cDNA templates were generated by SuperscriptIII First Strand Synthesis System (Thermofisher, Carlsbad, CA, United States), followed by eight separate nested PCR reactions; these eight amplicons span the near-whole HIV genome. Final amplicon products were sequenced by Sanger using the 3100 Genetic Analyzer (Applied Biosystems, Foster City, CA, United States) and by NGS using Nextera XT DNA Library Prep chemistry (Illumina, San Diego, CA, United States) to generate multiplexed libraries for Illumina’s MiSeq platform with 250 base paired-end reads. Sanger consensus sequences were generated manually using Sequencher version 5.2.4 (Gene Codes, Ann Arbor, MI, United States) to confirm degenerate nucleotides. NGS data were processed and demultiplexed using BaseSpace cloud application (Illumina, San Diego, CA, United States). NGS consensus sequences were called at a 20% threshold.

### Profile Sampling

**FIGURE 1:**
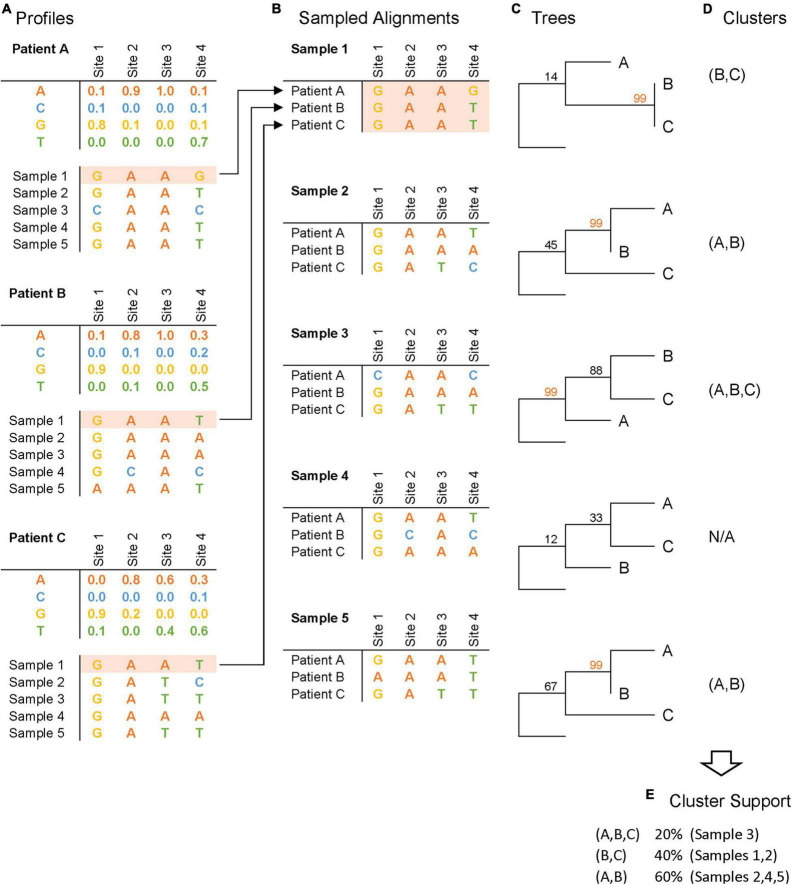
Profile sampling pipeline. This schematic figure depicts the four steps of the *profile sampling* process, illustrated here with 5 samples per patient: **(A)** NGS-derived frequencies at each HIV genome site for each patient are generated, and synthetic sequences are sampled from these frequency tables to summarize intra-host variation; **(B)** sampled sequences are collated across patients to construct sampled alignments; **(C)** phylogenetic trees are inferred with bootstrap support from the alignments; **(D)** clusters are inferred based on phylogenetic bootstrap support (illustrated here with bootstrap support ≥ 99); and **(E)** cluster support is measured as the frequency that a cluster is inferred across samples.

We introduce a new approach for incorporating within-host viral variation into phylogenetic analysis, called *profile sampling* (Figure 1). *Profile sampling* builds upon existing methods of phylogenetic and cluster inference by also sampling from within-host viral diversity. We start by aligning each individual’s HIV NGS reads using the hivmmer pipeline (Howison et al., 2019), which we developed and now extended to support near-whole-genome HIV data and perform codon-aware alignment within each gene (hivmmer version 0.2.1). A key feature of this pipeline is its use of profile hidden Markov models (HMMs) to model and align collections of HIV sequences. Profile HMMs have been abundantly used for biological sequence analyses and are particularly well-suited to modeling variation in populations of sequences (Eddy, 2004). Briefly, hivmmer performs quality control and error correction in overlapping regions of read pairs using PEAR version 0.9.11 (Zhang et al., 2014), translates them into possible reading frames, aligns them in amino acid space to profile HMMs of all HIV-1 group M reference sequences (Los Alamos National Lab, 2020) using the profile HMM alignment tool HMMER version 3.1b2 (Eddy, 2011), and produces a codon frequency table across the near-whole HIV genome. We refer to this resulting codon frequency table as the individual’s HIV *profile*.

Subsequently, we sample 500 fully-resolved sequences from each of the 37 individuals’ HIV profile according to the frequency of observed codons in the profile, for a total of 37 × 500 = 18,500 *profile-sampled* sequences. These sequences do not correspond to real strains present in the biological sample, but do capture the empirical distribution of within-host variation at the individual codon level. We note that the sequences do not capture linkage across codons, which is important for the detection and elimination of recombinant HIV strains as part of quality control, but is unessential for phylogenetic analyses. We then collate the 18,500 sequences into 500 *profile-sampled* data sets, by sampling without replacement so that each data set has 37 sequences (one for each individual) and can be used in a phylogenetic analysis with existing methods.

We also use the 18,500 sequences to estimate the within-host diversity for the 37 individuals as the average percent difference in nucleotides across all pairwise comparisons of each individual’s 500 *profile-sampled* nucleotide sequences. These pairwise differences are calculated using the Hamming distance (Allam et al., 2011) [also called **p**-distance (Maldarelli et al., 2013; Hassan et al., 2017)].

### Phylogenetic Inference

For *profile sampling*, we perform phylogenetic inference of wgs (HXB2 positions 790-9417) on each of the 500 *profile-sampled* data sets by estimating a multiple sequence alignment with OMM_MACSE version 10.02 (Ranwez et al., 2018) and a maximum-likelihood phylogeny with the GTRCAT model and 100 rapid bootstrap replicates using RAxML version 8.2.12 (Stamatakis, 2014), with HIV-1 group O (GenBank accession L20587.1) as the outgroup. We perform this same phylogenetic inference on three clinically-relevant sub-genomic regions: protease and reverse transcriptase at the beginning of the *pol* gene (“prrt”, HXB2 positions 2253-3554), *int* gene (HXB2 positions 4230-5096), and *env* gene (HXB2 positions 6225-8790). The prrt and *int* regions are routinely sequenced in clinical care to detect drug resistance and inform clinical anti-retroviral therapy choices. The *env* region is sequenced to genotypically infer viral tropism and co-receptor usage. Cluster inference is performed on all phylogenies using Cluster Picker (Ragonnet-Cronin et al., 2013) with a threshold of 99% bootstrap support.

In addition to *profile sampling*, we infer phylogenies with similar tools, regions and parameters for the NGS consensus sequences at the 20% threshold and the Sanger sequences. We perform cluster inference on all consensus phylogenies using a similar method as for *profile sampling*. We do not impose a genetic distance threshold because empirically-justified thresholds that are comparable across the near-whole-genome and the *int* and *env* regions have not to our knowledge been established. This approach of using only bootstrap criteria for cluster detection is consistent with methods commonly used in the broader HIV cluster analysis literature (Hassan et al., 2017).

We investigate the impact of within-host diversity on phylogenetic topology and evolutionary distance estimates in the sub-genomic and near-whole-genome regions. To examine variation in topology, we first calculate pairwise geodesic distance (Billera et al., 2001; Owen and Provan, 2011) among the 500 phylogenies from the profile samples, as well as phylogenies from the NGS consensus and Sanger sequences. Then we perform multi-dimensional scaling on the resulting distance matrix to visualize topological space in two dimensions. Next, to examine variation in estimated evolutionary distance, we sum the branch lengths within each phylogeny across all branches and across only tip branches and visualize the distribution of these branch length sums. These analyses establish to what extent phylogenies from consensus sequences (which are point estimates) summarize the underlying variation in two important aspects of the phylogeny: estimated topology and estimated evolutionary distance.

Finally, we examine the clusters that are detected in phylogenies of NGS consensus sequences versus Sanger sequences, and across the four genomic regions and their *profile sampling* support, using the frequency that a cluster appears across the 500 *profile-sampled* phylogenies. We refer to this value as the *profile-sampled* support and note that it can be conceived as analogical to the conventional bootstrap support for evaluating robustness of an individual phylogeny’s topology but extends that feature to evaluating robustness of cluster detection using within-host sequence variation.

All analysis source code is available from https://github.com/kantorlab/hiv-profile-sampling.

## Results

### Profile Sampling Estimates of Within-Host Diversity

**FIGURE 2:**
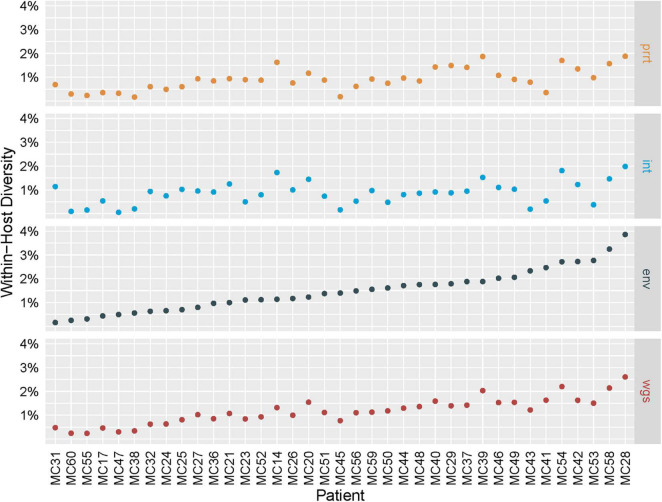
Intra-host genetic diversity by genomic region. Intra-host genetic diversity (*Y* axis; defined as the average percent difference across all pairwise comparisons of the 500 *profile-sampled* nucleotide sequences for an individual) of the four examined genomic regions (gray boxes on the right) in the 37 sampled individuals (*X* axis) is highest in *env* for most individuals and lies within the range of previously reported values.

Figure 2 shows the estimated within-host percent diversity in each examined genomic region across individuals, ordered by *env*, which we expected *a priori* to be the most variable region. The largest estimated diversity is in *env* for individual MC28 (3.9%), and *env* has the overall largest estimated diversity range (0.2–3.9%, mean 1.5%). The other regions have ranges of 0.2–1.9% (mean 0.9%) for prrt, 0.1–2.0% (mean 0.9%) for *int*, and 0.2–2.6% (mean 1.2%) for wgs. Such within-host estimations are not feasible with conventional consensus Sanger or NGS approaches, although methods such as phyloscanner and HAPHPIPE that utilize deeper NGS sequencing to build phylogenies with multiple tips per sample, are able to also quantify within-host diversity (Wymant et al., 2018; Bendall et al., 2021).

### Phylogenetic Estimates Are Sensitive to Within-Host Diversity

**FIGURE 3:**
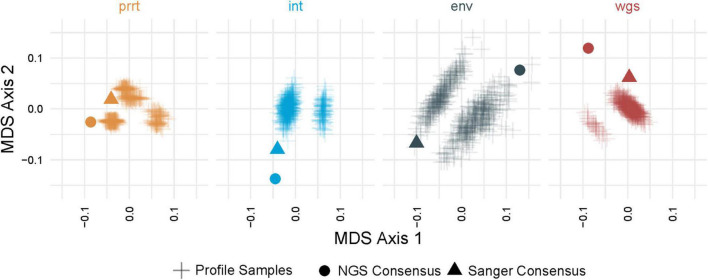
Multi-dimensional scaling (MDS) of pairwise geodesic distance among maximum-likelihood phylogenies from the *profile sampling* approach within genomic regions. MDS Axis 1 (*X* axis) and Axis 2 (*Y* axis) show that the space of inferred phylogenies is multi-modal for all genomic regions. The phylogenies from NGS and Sanger consensus sequences (dot and triangle) are point estimates that do not capture the full variation in phylogenies that can be inferred from deeply-sequenced NGS data (plus signs) in all examined genomic regions (colors).

**FIGURE 4:**
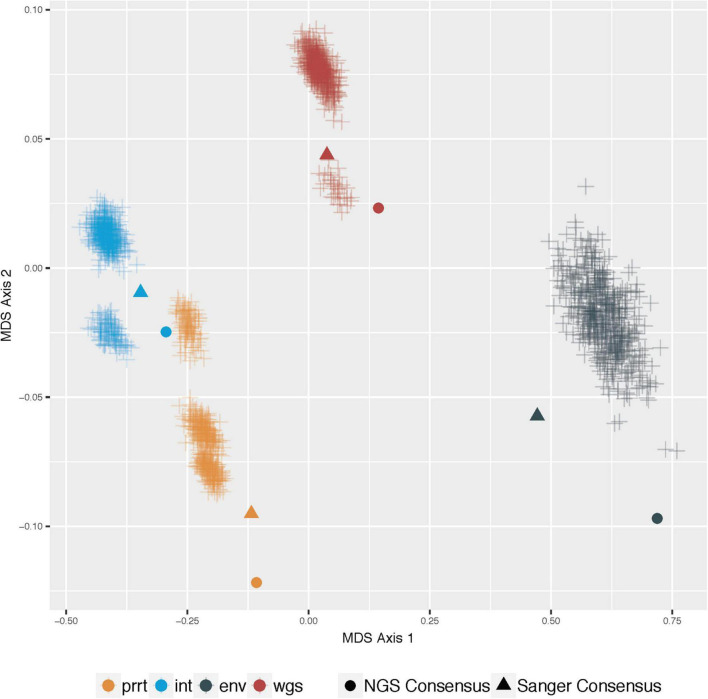
Multi-dimensional scaling (MDS) of pairwise geodesic distance among maximum-likelihood phylogenies from the *profile-sampling* approach across all genomic regions. MDS Axis 1 (*X* axis) and Axis 2 (*Y* axis) show that the space of inferred phylogenies is multi-modal for all genomic regions. The phylogenies from consensus sequences (dot and triangle) are point estimates that do not capture the full variation in phylogenies that can be inferred from deeply-sequenced NGS data (plus signs) in all examined genomic regions (colors).

Figure 3 demonstrates multi-dimensional scaling on the *profile-sampled* phylogenies and the phylogenies from Sanger and NGS consensus sequences *within* each genomic region. The *profile sampling* approach reveals for each genomic region a multi-modal topological space in which phylogenies inferred from both Sanger and NGS consensus sequences are outliers; a result that is confirmed by multi-dimensional scaling *across* all regions (Figure 4). A key difference between the consensus and *profile-sampled* sequences is that consensus sequences contain ambiguous nucleotides at sites with ≥2 nucleotides by Sanger Sequencing base calling or with ≥20% frequency for NGS. In contrast, *profile-sampled* sequences by construction have no ambiguous sites, and ambiguity is instead incorporated into analyses through frequency of the ambiguous nucleotides across the 500 samples.

**FIGURE 5:**
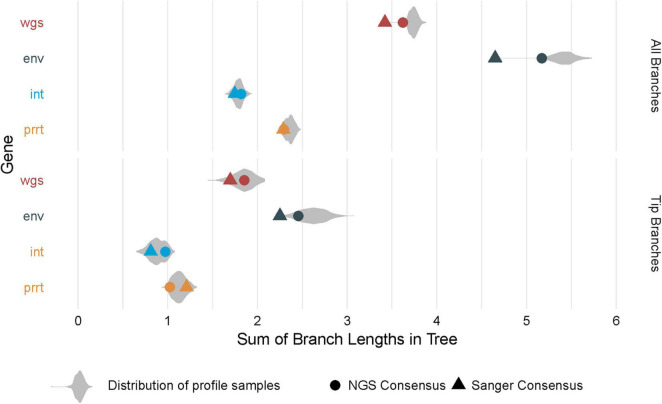
Distribution of branch length sums across phylogenies. The figure demonstrates total branch lengths (*X* axis), in each of the *profile-sampled* phylogenies (*Y* axis and colors). The phylogenies from consensus sequences (dot and triangle) can lie at extreme values within these distributions, both when considering the lengths across all branches (top) and the lengths across only the branches at the tips (bottom).

Figure 5 shows the distribution of branch length sums across compared phylogenies. Overall, estimates are larger in *env* and wgs, and smaller when restricting to only tip branches. In some cases, consensus phylogenies provide an adequate summary of the distribution (as in the phylogeny of the NGS consensus sequence for tip branches for wgs). In other cases, consensus phylogenies have estimates that are outliers in the distribution (as in the phylogenies from NGS and Sanger consensus sequences for all branches in wgs and *env*).

Taken together, the heterogeneity between the phylogenetic results from *profile sampling* and consensus-inferred point estimates demonstrate that within-host virus sequence diversity impacts the inference of virus phylogeny across individuals, and that the consensus approach to handling ambiguity and collapsing within-host sequence variation can obscure both the magnitude and effect of these impacts.

### *Profile-Sampled* Cluster Support Differs by Sequencing Depth and Genomic Region

**FIGURE 6:**
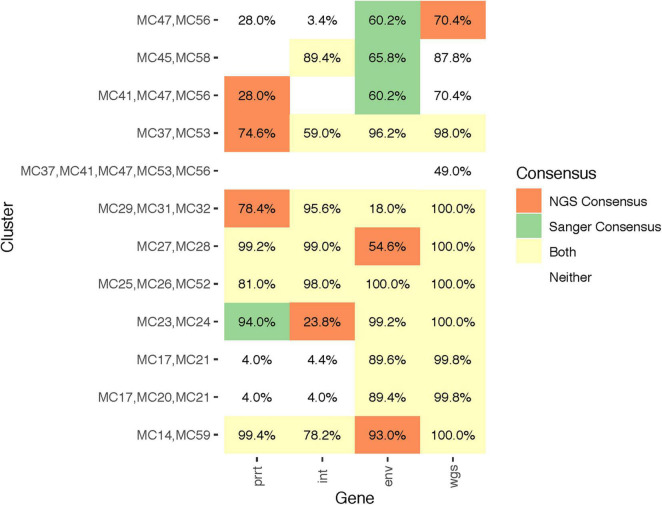
Quantitative differences in *profile-sampled* cluster support across genomic regions. The figure illustrates the clusters and their subclusters (*Y* axis) identified by Sanger versus NGS consensus sequences (colors; see legend) across genomic regions (*X* axis). Numeric values indicate cluster support from the *profile sampling* method. A blank cell indicates that the cluster was not detected in that genomic region.

Combining the results of all examined methods (Sanger, NGS consensus, NGS *profile sampling*) and genomic regions (prrt, int, env, wgs) there were overall 12 identified clusters among the 37 participants. Seven clusters had two members, four had three members, and one had five members. Figure 6 demonstrates comparison of cluster detection by examined methods and genomic regions. Some clusters had consistently high support (>75%) across all regions (e.g., MC25/MC26/MC52 and MC14/MC59). Other clusters had higher support in certain regions (e.g., MC17/MC20/MC21 in *env* and wgs). Eight clusters across different regions were detected by *profile sampling* but not by consensus methods, while all clusters detected by consensus methods were detected by *profile sampling*. One larger cluster, MC37/MC41/MC47/MC53/MC56, was detected only by *profile sampling* with the wgs dataset.

By providing previously-unavailable cluster support that considers within-host “deep” viral variation, *profile sampling* in the wgs dataset allowed detection of the largest (all 12) overall number of clusters. The clusters detected in wgs also had the highest overall *profile-sampled* support, as compared to the other genomic regions. The median *profile-sampled* support was 99.8% for wgs, 77.6% for *env*, 41.4% for *int*, and 51.3% for prrt. The *profile-sampled* support for wgs was significantly larger than for *int* (*p*-value = 0.005, Dunn’s test of multiple comparisons using paired rank sums with Holm-Bonferroni correction) and prrt (*p*-value = 0.010), but not significantly larger than for *env* (*p*-value = 0.092).

**FIGURE 7:**
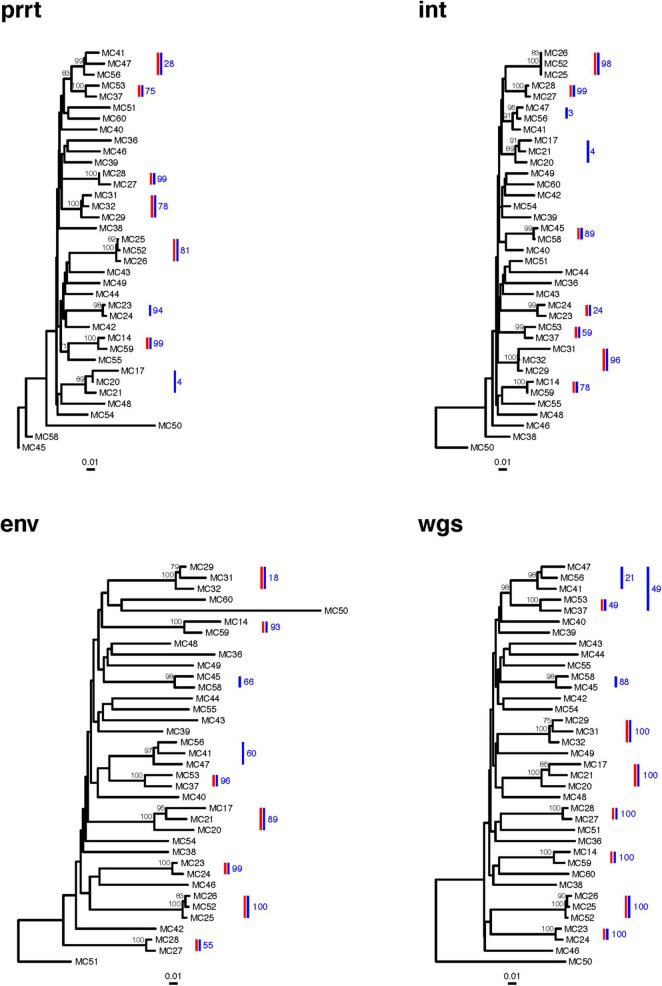
Next-generation sequencing (NGS) consensus sequence phylogenetic trees of 37 new HIV diagnoses in RI according to genomic region. The figure demonstrates clusters in phylogenetic trees from four genomic regions (prrt-protease reverse transcriptase; int-integrase; env-envelope; wgs-whole genome sequence). Clusters (≥99% bootstrap support) inferred from the phylogenies of NGS consensus sequences (vertical red bars) differ across genomic regions. The largest number of clusters was inferred from *int, env*, and wgs, and the smallest number from prrt. *Profile sampling* detected additional clusters (vertical blue bars) and provided a bootstrap-like measure of cluster support (annotation to blue bars). Bootstraps > 70% are shown to the left of the relevant node. Trees are rooted by an HIV-1 group O sequence, which is omitted from the plots.

**FIGURE 8:**
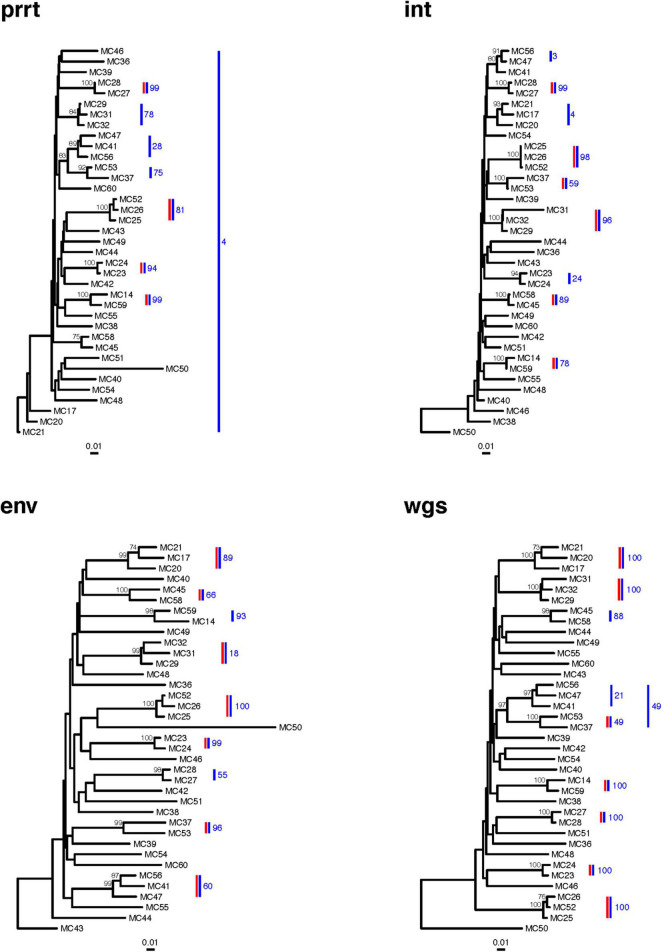
Sanger sequence phylogenetic trees of 37 new HIV diagnoses in RI according to genomic region. The figure demonstrates clusters in phylogenetic trees from four genomic regions (prrt-protease reverse transcriptase; int-integrase; env-envelope; wgs-whole genome sequence). Clusters (≥99% bootstrap support) inferred from the phylogenies of Sanger consensus sequences (vertical red bars) differ across genomic regions. The largest number of clusters was inferred from env and wgs, and the smallest number from prrt. *Profile sampling* detected additional clusters (vertical blue bars) and provided a bootstrap-like measure of cluster support (annotation to blue bars). Bootstraps > 70% are shown to the left of the relevant node. Trees are rooted by an HIV-1 group O sequence, which is omitted from the plots.

The phylogenies of NGS consensus sequences detected only six clusters in prrt, seven in *int*, seven in *env*, and seven in wgs (Figure 7). The phylogenies of Sanger sequences detected only four clusters in prrt, six in *int*, seven in *env*, and seven in wgs (Figure 8). Only one cluster (MC25/MC26/MC52) was consistently detected across phylogenies from NGS and Sanger consensus sequences, and across all regions.

The median *profile-sampled* support for clusters detected by Sanger consensus sequences (green and yellow cells, Figure 6) was 60.2%, not different than for those detected by NGS consensus sequences (72.5%; orange and yellow cells in Figure 6; *p*-value = 0.415, Wilcoxon signed-rank test). Totaling the clusters detected across the four regions, phylogenies of Sanger consensus sequences detected fewer clusters (27) than phylogenies of NGS consensus sequences (31) or *profile sampling* (43); and detected fewer clusters in each region except *env*. Cluster support values were higher for clusters detected by phylogenies of both NGS and Sanger sequences (yellow cells, Figure 6; median cluster support 98.5%) than those detected only by one or the other (orange or green cells, Figure 6; median cluster support 68.1%).

## Discussion

Current phylogenetic approaches to inference of HIV transmission clusters utilize consensus sequences to summarize within-host sequence variation. We introduce a different summarization strategy, *profile sampling*, that preserves the within-host sequence variation provided by the deeper sequencing that is now widely available. In a dataset of all newly HIV-diagnosed individuals over six months at the largest HIV center in Rhode Island, United States, deeper sequencing provided by NGS and incorporated by the newly-introduced *profile sampling* captured within-host diversity, revealing clusters detected by *profile sampling* but not by consensus approaches, including one larger cluster found only with *profile sampling* of the wgs. This suggests that routinely used consensus sequence approaches discard potentially relevant information present in NGS data, and that considering this additional information in phylogenetic analysis may improve the robustness of HIV cluster detection. *Profile sampling* can thus provide a new quantitative measure of cluster confidence with potential applications to public health activities. Such activities could be better justified in scenarios where clusters triggering them have high cluster support from deep-sequenced data, though this was not addressed here and needs to be demonstrated.

*Profile sampling* complements well-established bootstrapping methods, and in some senses is orthogonal to them. Phylogenetic inference depends on a sequence alignment, where each row corresponds to a single host and each column corresponds to a given genomic position. Bootstrapping resamples columns of the matrix with replacement, giving an indication of how consistent signal is across genomic positions. *Profile sampling*, on the other hand, resamples each site in the alignment given the sequence diversity observed within each host. This gives an indication of how consistent signal is across HIV genomes within each host. Variation in within-host diversity could be due to a variety of biological factors, like viral mutation, effective viral population size, and time since infection, as well as technical factors like sequencing depth and sequencing errors. Not accommodating this variation could lead to overconfidence in results or missed clinically relevant phylogenetic signals.

Although the standard practice of collapsing within-host diversity into a single consensus sequence simplifies downstream analyses, the results presented here demonstrate that this practice discards potentially relevant biological results and may mislead phylogenetic analysis and resulting epidemiological consequences. For example, public health activities triggered by phylogenetic inference of HIV molecular clustering to inform and improve prevention and treatment interventions can be affected (Peters et al., 2016). In our data, clusters vary in their *profile-sampled* support, and consensus approaches can fail to detect clusters supported by deep-sequenced data, as in the case of the largest cluster, which was detected by *profile sampling*, not by consensus approaches. As data acquisition increasingly shifts to NGS approaches (Kantor, 2021), it is important to compare results of larger datasets from new methods to the more common conventional Sanger *pol* consensus sequences.

Much of the enthusiasm about shifting from Sanger sequencing to NGS has been due to reducing costs and the ability to more easily collect data on the entire HIV genome rather than few genes. Our results suggest that much benefit from NGS may also come from its greater sequencing depth, capturing viral sequence variation within individuals. This benefit can only be realized, though, if this variation is propagated to phylogenetic analyses, such as by *profile sampling* introduced here, rather than being collapsed to a consensus sequence, as is conventionally done. We suggest creating a profile that captures that variation, performing multiple phylogenetic analyses on sequences sampled from the profile, and then summarizing the phylogenetic analyses. This summary method can also take advantage of the output from long-read sequencing technologies which are able to provide fully resolved sequences from the viral population and which are starting to replace short-read NGS sequencing in a number of HIV labs. Future tools could incorporate the variation directly into the phylogenetic inference process itself (Leitner, 2019).

In our comparison of cluster inference across genomic regions, we found that fewer clusters were detected overall in prrt and *int* compared to *env* and wgs. Prior studies of clustering from Sanger consensus sequences present mixed results on prevalence of clustering across genomic regions. Some studies found concordant clustering across *gag*-*env* (Han et al., 2009) and *gag*-*pol*-*env* (Kaye et al., 2008; English et al., 2011), while others found fewer clusters in *pol* than in *env* (Kapaata et al., 2013), or in *gag*-*env* than in *pol* (Ndiaye et al., 2013). The additional information available in deep-sequenced NGS data, along with cluster support measures provided by *profile sampling*, could help resolve differences, as suggested here. In our data, not only were more clusters detected in the near-whole length genome, but those clusters also had higher cluster support as measured by deep-sequenced NGS data. While prior studies demonstrated better accuracy of cluster inference on simulated NGS sequences when using wgs (Yebra et al., 2016) and that the proportion of Sanger sequences in clusters increased with longer sequence regions (Novitsky et al., 2015), we have demonstrated here that deeply-sequenced, near-whole length NGS data can be used with *profile sampling* to detect clusters undetectable by consensus approaches. The impact of this approach for public health remains to be determined.

One limitation of our study is the small number of participants and the short timeframe they were enrolled in. However, participants represent a dense temporal sampling, and comprise all newly HIV-diagnosed individuals in a six-month period at the largest HIV center in Rhode Island, in which 80% of the state’s people with HIV are cared for. The overall size of the HIV epidemic in Rhode Island was estimated as 2,396 individuals in 2016 (Rhode Island Department of Health, 2019), but NGS data for this population are not currently available beyond those presented here. In future work, we will apply *profile sampling* to larger NGS data sets, to assess cluster inference concordance between Sanger and NGS data, and its impact on public health actions to halt HIV transmission. Additionally, we do not know the real number of clusters or the true transmission chains, a limitation with all studies on HIV transmission networks. Our construction of HIV profiles from NGS data is also limited by the accuracy of the NGS assays themselves. The codon frequencies in the profiles may be biased measures of the true within-host diversity because of biases in PCR amplification, as well as a variety of technical factors related to next-generation sequencing and analysis [see Howison et al. (2019) for a detailed discussion]. Sequencing protocols such as Primer ID (Jabara et al., 2011; Zhou et al., 2020) have been introduced to reduce and correct for these biases, and should be considered in the future.

In conclusion, the true HIV transmission network is unknown, but phylogenetic analysis and cluster inference are promising tools for aiding clinicians and public health officials in better understanding and in disrupting HIV transmission (Fauci et al., 2019). Most current phylogenetic approaches do not fully utilize the information on within-host diversity available in deep-sequenced, near-whole-genome NGS data. As NGS data sets are increasingly available and become more representative of HIV epidemics, we suggest that the additional information they measure has the potential to improve the robustness of HIV molecular cluster inference, the impact of which needs to be further investigated.

## Data Availability Statement

The datasets presented in this article are not readily available because of patient privacy concerns. Requests to access the datasets should be directed to RK at rkantor@brown.edu.

## Ethics Statement

The studies involving human participants were reviewed and approved by Institutional Review Board at Lifespan. The patients/participants provided their written informed consent to participate in this study.

## Author Contributions

AG, MH, PC, CD, and RK contributed to the conception of the study. LL, MD’A, PC, and RK contributed to data collection and organization. AG and CL created the method. AG and MH contributed to implementation, analysis and visualization. AG, MH, CD, and RK contributed to writing the manuscript. All authors contributed to manuscript revision, read, and approved the submitted version.

## Conflict of Interest

The authors declare that the research was conducted in the absence of any commercial or financial relationships that could be construed as a potential conflict of interest.

## Publisher’s Note

All claims expressed in this article are solely those of the authors and do not necessarily represent those of their affiliated organizations, or those of the publisher, the editors and the reviewers. Any product that may be evaluated in this article, or claim that may be made by its manufacturer, is not guaranteed or endorsed by the publisher.
